# Functionality and Quality of Asthma mHealth Apps and Their Consistency With International Guidelines: Protocol for a Systematic Search and Evaluation of Mobile Apps

**DOI:** 10.2196/33103

**Published:** 2022-02-09

**Authors:** Billy Robinson, Enying Gong, Brian Oldenburg, Katharine See

**Affiliations:** 1 Department of Respiratory Medicine Northern Health Epping Australia; 2 Nossal Institute for Global Health University of Melbourne Melbourne Australia; 3 Noncommunicable Disease Control Unit University of Melbourne Melbourne Australia; 4 World Health Organization Collaborating Centre on Implementation Research for Prevention and Control of Noncommunicable Diseases World Health Organization Melbourne Australia

**Keywords:** asthma, mHealth, mobile phone, applications, self-management, chronic disease, respiratory, smartphone, asthma app

## Abstract

**Background:**

Asthma is a chronic respiratory disorder that requires long-term pharmacotherapy and patient empowerment to manage the condition and recognize and respond to asthma exacerbations. Mobile health (mHealth) apps represent a potential medium through which patients can improve their ability to self-manage their asthma. Few studies have conducted a systematic evaluation of asthma mobile apps for quality and functionality using a validated tool. None of these reviews have systematically assessed these apps for their content and evaluated them against the available international best practice guidelines.

**Objective:**

The objective of this study is to conduct a systematic search and evaluation of adult-targeted asthma mHealth apps. As part of this review, the potential of an mHealth app to improve asthma self-management and the overall quality of the app will be evaluated using the Mobile App Rating Scale framework, and the quality of the information within an app will be evaluated using the current Global Initiative for Asthma guidelines as a reference.

**Methods:**

A stepwise methodological approach was taken in creating this review. First, the most recent Global Initiative for Asthma guidelines were independently reviewed by 2 authors to identify key recommendations that could be feasibly incorporated into an mHealth app. A previously developed asthma assessment framework was identified and was modified to suit our research and ensure that all of these identified recommendations were included. In total, 2 popular app stores were reviewed to identify potential mHealth apps. These apps were screened based on predefined inclusion and exclusion criteria. Suitable apps were then evaluated. Technical information was obtained from publicly available information. The next step was to perform an app quality assessment using the validated Mobile App Rating Scale framework to objectively determine the quality of an app. App functionality was assessed using the Intercontinental Medical Statistics Institute for Health Informatics Functionality Scoring System. Finally, the mHealth apps were assessed using our developed checklist.

**Results:**

Funding has been received for the project from the Respiratory Department at Northern Health, Victoria. Three reviewers have been recruited to systematically evaluate the apps. The results of this study are expected in 2022.

**Conclusions:**

To our knowledge, this review represents the first study to examine all mHealth apps available in Australia that are targeted to adults with asthma for their functionality, quality, and consistency with international best practice guidelines. Although this review will only be conducted on mHealth apps available in Australia, many apps are available worldwide; thus, this study should be largely generalizable to other English-speaking regions and users. The results of this review will help to fill gaps in the literature and assist clinicians in providing evidence-based advice to patients wishing to use mHealth apps as part of their asthma self-management.

**Trial Registration:**

PROSPERO 269894; https://www.crd.york.ac.uk/prospero/display_record.php?RecordID=269894

**International Registered Report Identifier (IRRID):**

PRR1-10.2196/33103

## Introduction

### Background

Asthma is a chronic respiratory disorder that is clinically defined as a combination of typical respiratory symptoms (outlined below) and significant variable reversible airflow limitation [1]. Symptoms that people with asthma can experience include periods of shortness of breath, cough, wheezing, and chest tightness. When the frequency or severity of these symptoms increases compared with a patient’s baseline respiratory status, it represents an *asthma exacerbation* or *flare-up* [2].

Asthma is a significant worldwide chronic health issue affecting 1% to 18% of the global population [3]. In Australia, 2.7 million people have asthma, representing 11% of the total population [4]. A 2012 survey of Australians with asthma found that 10% of them had presented to an emergency department ≥1 time for asthma-related symptoms, and 29% reported an urgent health care visit (to either a general practitioner or emergency facility) [4]. Asthma accounts for 34% of Australia’s burden of disease because of respiratory conditions and 2.5% of Australia’s total burden of disease [4]. Australian adults with asthma are more likely to describe themselves as having a poor quality of life compared with those without asthma and are less likely to rate their health status as excellent or very good. This trend is more pronounced among those with severe or poorly controlled asthma [4]. When observing the total cost that asthma has on the Australian health system, it is evident that hospital-related costs outweigh non–hospital-related costs (Aus $205 million/year [approximately US $150 million] vs Aus $163 million/year [approximately US $120 million]) [4]. Theoretically, reducing exacerbations would reduce the requirement for hospitalizations, unplanned primary care presentations, and indirect costs such as work absenteeism, and thus assist in bringing these costs down.

The Global Initiative for Asthma (GINA) guidelines represent regularly updated guidelines based on reviews of the available scientific literature by an international panel of experts [3]. It is from these guidelines that many local asthma management guidelines stem from. In addition to pharmacotherapy for asthma management, the GINA guidelines advise that a key aspect of treatment is educating patients on recognizing symptoms of asthma exacerbations and learning when and how to self-manage them [3]. Mobile health (mHealth) apps represent a potential medium through which patients can improve their ability to self-manage their asthma. From the most recent Deloitte [5] review of Australia’s telecommunication status, 89% of the Australian population uses smartphones. This widespread, almost ubiquitous use of smartphones, and the apps that run on them, represents an opportunity to empower patients to track asthma symptoms, learn about their condition, and undertake practical self-management strategies. A number of systematic evaluations of asthma mobile apps have been conducted; however, to our knowledge, none have assessed all available apps for the presence and quality of information that they provide compared with available best practice management guidelines in a systematic way [6-8].

This review will look at both free and paid asthma mHealth apps targeted toward adults with asthma available from the Apple App Store (iOS) and Google Play Store (Android) and examine the functionality and quality of these apps and the consistency of these available apps with recommendations from the GINA guidelines, making it the first review of its kind. All of the review processes will be conducted on apps available in the region of the researchers, Australia. Although mobile apps are often published across multiple regions in the same language, different regions can have different apps available on their stores. As such, some identified mHealth apps may not be available in all regions, whereas others available in other regions may not be available in Australia. However, given that most apps identified in this review will also be available in other English-speaking regions, the results should be largely generalizable to these regions.

### Objective

The objective of this study is to conduct a systematic search and evaluation of the available English-language mHealth apps targeted to adults with asthma, assess their potential for improving asthma self-management, and assess the quality of the information they provide using the current GINA guidelines as a reference.

## Methods

### Overview

The methodology of how we will achieve these research objectives is explained in this section. First, the GINA guidelines were read by 2 medical professionals (BR and KS) to identify and establish a consensus of key recommendations in the guidelines that could be feasibly incorporated into an app for asthma management. Next, mobile apps in the selected app stores will be identified. After identification, we will screen the apps based on the selection criteria. Finally, we will evaluate the quality and functionality of the mHealth apps and extract this information into a database.

### Study Setting

This study will be conducted in Australia by medical practitioners, medical students, and digital health researchers. It will assess mHealth apps presented in English on Australian mobile app stores. Given that the clinicians involved in this research are adult physicians, and the management principles of asthma vary significantly between adult and pediatric populations, only those mHealth apps targeted toward adults with asthma will be evaluated. Where possible, we have followed the PRISMA (Preferred Reporting Items for Systematic Reviews and Meta-Analyses) guidelines for systematic reviews [9]. Given that this is a protocol and is a review of mobile apps instead of journal articles, some of the items in the guideline checklist are not relevant to this protocol.

### Review of the GINA Guidelines to Establish That the Recommendations Are Feasible for Incorporation Into an mHealth App

We will use the established Mobile App Rating Scale (MARS) to assess the usability and overall quality of an app, as detailed in the section below. Although this framework is helpful for reviewing general app features and quality, it does not possess information specific to asthma management. There is no validated peer-reviewed asthma mHealth app checklist or framework. However, a previously developed asthma app assessment framework from Guan et al [10] has been created by researchers in China. The framework developed by these researchers has not been derived and validated into an instrument since its production; however, it was developed in a systematic fashion using a Delphi survey technique and by consulting a number of different respiratory specialists [10]. Although this represents a solid and well–thought-out framework, there were a number of issues identified with only using this framework for our study. First, it includes a number of topics regarding the utility and usability of an app. For our study, we have elected to use the validated MARS assessment framework to look at these areas. Thus, looking at these topics doubles the information that is already being looked at. Second, we wanted to ensure that the recommendations identified from the GINA guidelines were in the checklist. Although the framework by Guan et al [10] has been developed by numerous respiratory specialists, it never specifically references these global guidelines in its development. We want to ensure current best practice guidelines are met and thus have used these guidelines. Third, given that this checklist was developed by overseas practitioners, we wanted to ensure that it was still applicable to Australian and Western clinicians and patients. Finally, this is not a validated tool, and as such, caution is warranted when using this framework. For these reasons, we sought to have 2 clinicians review the current international best practice asthma guidelines to establish which recommendations could be feasibly incorporated into an mHealth app and develop our own checklist, which we will meld with aspects of the framework by Guan et al [10].

This was conducted independently by 2 of the primary team members: the first and final authors. The 2020 GINA guidelines were read in full, and each reviewer noted recommendations from the GINA that they believed could be incorporated into an asthma app for patients. We hoped to capture all relevant recommendations that could feasibly be integrated into a mobile app by conducting this process in an independent manner with reviewers at different stages in their respiratory medicine careers. After each author had identified their recommendations, they came together to see if a consensus could be reached on them. If no consensus could be reached, the plan was for an independent senior member of the team to review the identified recommendations to make a decision. However, this was not required. The authors either identified the same or similar recommendations or agreed with the recommendations that the other authors identified that they did not. The identified recommendations from each author and the recommendations where a consensus was reached, which represent the final identified recommendations, are shown in Table 1.

Therefore, we will develop a checklist from these identified recommendations and a modified version of the framework by Guan et al [10] to ensure that all of our recognized recommendations are included and that the information we intend to obtain using other measures (ie, the MARS framework) is not. This checklist will be demonstrated later in this protocol.

The presence or absence of the features derived from the checklist by Guan et al [10] and the GINA guidelines will be used as a marker of the information quality of the app. Given that we are modifying their checklist, we will not assign the weighting that Guan et al [10] attributed to certain framework groups. Furthermore, given that these apps are for use by patients with asthma and not health professionals, what a patient considers important may, and often does, differ from their health professional. Thus, the weighting does not hold as much relevance in our study as the subgroups have been weighted by physician impression of importance. Therefore, our checklist only examines whether an asthma app does or does not contain information or features developed using the GINA guidelines or the framework by Guan et al [10]. We will assess these apps using both our developed checklist and the MARS framework.

**Table 1:** Recommendations identified from the Global Initiative for Asthma guidelines that could feasibly be incorporated into a mobile health app.

Reviewer 1	Reviewer 2	Consensus reached?
Assess symptom control (eg, ACQ^a^)	Support for assessing symptom control over a 4-week period	Support for assessing symptom control over a 4-week period looking at the frequency of asthma symptoms, night waking because of asthma, frequency of SABA^b^ use, and any activity limitation because of asthma; uses recognized screening, symptom control or numerical asthma control tools, and peak flow measurement
Ability to self-track symptoms with or without peak flow	—^c^	Encourages patients to track symptoms and peak flow measurements
Risk factors for future exacerbations	Helps users identify the future risk of exacerbations	Helps users identify the risk of future exacerbations
Screening for comorbidities and education regarding managing them	Screens for comorbidities and assists patients with managing them	Screens for relevant comorbidities and educates patients on the management of these
Inhaler technique with or without video	Provides education on appropriate inhaler techniques	Provides education on appropriate inhaler techniques with visual aids
Ability to record action plan	Provides an area for patients to keep and refer to their written action plan	Provides an area for patients to keep and refer to their written action plan
Reminder to engage with primary care	Reminds users to see their health care provider for management and review of their asthma	Provides reminders to users to see their health care provider for management and review of their asthma
—	Specifically provides the suggestion to see a health care provider if a patient is using a SABA alone	Specifically provides the suggestion to see a health care provider if a patient is using a SABA alone
Medication adherence	Prompts users to adhere to controller medications even when symptoms are infrequent	Prompts users to adhere to controller medications even when symptoms are infrequent
General asthma education	Provides knowledge on general asthma information, management of asthma, modifiable risk factors and strategies to address them, and when to see a health care provider	Provides knowledge on general asthma information, management of asthma, modifiable risk factors and strategies to address them, when to see a health care provider, and identification and management of comorbidities
Help with activating action plan	Provides advice on when to refer to a patient’s asthma action plan based on self-monitoring of symptoms or PEF^d^	Provides advice on when to refer to a patient’s asthma action plan based on self-monitoring of symptoms or PEF
—	Prompts patient to see primary HCP^e^ if features of an asthma exacerbation (symptoms and SABA use) are identified via the app	Prompts patient to see primary HCP if features of an asthma exacerbation (symptoms and SABA use) are identified via the app

^a^ACQ: Asthma Control Questionnaire.

^b^SABA: short-acting *β*-agonist.

^c^Not available. Recommendation identified by one reviewer but not the other.

^d^PEF: peak expiratory flow.

^e^HCP: health care provider.

### Identification, Screening, and Selection of Mobile Apps for Review

This review will include both free and paid apps from the two most popular app stores in Australia across iOS and Android operating systems: the Apple App Store and Google Play Store.

Our approach to identifying these apps will follow the approach used in similar studies [6-8]. We will use the search bar in each of the stores and input the term *asthma*. This is a broad search category that will yield a number of results, some irrelevant to our review. However, the point of this is to capture all apps for review. This search will occur on August 10, 2021, in Melbourne, Australia. At the time of publication, this has already occurred.

All reviewers will be instructed to ensure that their operating system is up to date before commencing the following steps.

In total, 2 reviewers will search both app stores independently on the same day. One of the reviewers will be the primary author, and the second is a final year medical student with interest in digital health. These are 2 of the 3 people who will review the apps later in the study. Having 2 independent reviewers will aid quality assurance at this point of the review and reduce the risk of selection bias. The primary author of the study will review these apps, as will a final year medical student who is yet to be recruited. Screenshots will be taken of the results pages and sent to the primary researcher for record keeping. After obtaining the results for this search term, each reviewer will input the information into a Microsoft Excel spreadsheet (see Multimedia Appendix 1 for an example) in the order that they appear in the search results. The reviewers will then compare their results to ensure that they have captured all the available apps. If an app has been missed, the reviewers will need to recheck the stores until they have identified all available apps.

Screening of the apps identified in the previous step will occur next. This process will be comparable with similar reviews that have looked at the quality of mobile apps for diabetes self-management [11]. For all apps identified above, the same 2 reviewers will individually look at the app title, description, and attached photos, looking to identify inclusion and exclusion criteria for the app to undergo further evaluation.

An identified asthma app will be included in the evaluation stage if all of the following apply: (1) its primary role is related to asthma, (2) it is targeted to those with asthma, (3) it can be run on mobile phones, and (4) it is in English.

Apps will be excluded if any of the following apply: (1) it is not primarily related to asthma, (2) it is primarily targeted toward health care professionals (as stated in the product description), (3) it is not in English, and (4) it is targeted toward pediatric patients.

This information will be entered into a Microsoft Excel spreadsheet for record keeping (Multimedia Appendix 2).

Next, all apps included and not excluded at this stage will be downloaded by a third reviewer. The purpose of this is to identify apps that do not install properly or function after downloading. In the event of a suspected app malfunction, the reviewer will alert another reviewer, who will also attempt to download and operate the app. The purpose of this is to eliminate the risk that it is just the individual reviewer’s phone that is malfunctioning. If both reviewers cannot properly install or get the app to function after download, it will be eliminated from the review.

If, at any stage up to this point, discrepancies exist between the reviewers where they cannot reach a consensus, the app will be reviewed by a senior team member to make the final decision.

Finally, a last round of screening will be conducted by a third member of the research team. Here, the following apps will be removed from the review:

1. Duplications (ie, those apps that are available from both stores)
  In the event that an app is available on both stores, the most updated version of the app will be kept.
  In the event of an app having the same update date on both stores, it will be left to the discretion of the reviewer as to which app of the 2 is included for further review.
2. Highly similar versions of the same app (eg, a pro version)
  In the event of this occurring, the pro version will be kept. This is as our primary goal is to assess for best-quality asthma apps.
3. Apps that are no longer available for download

**Figure 1:**
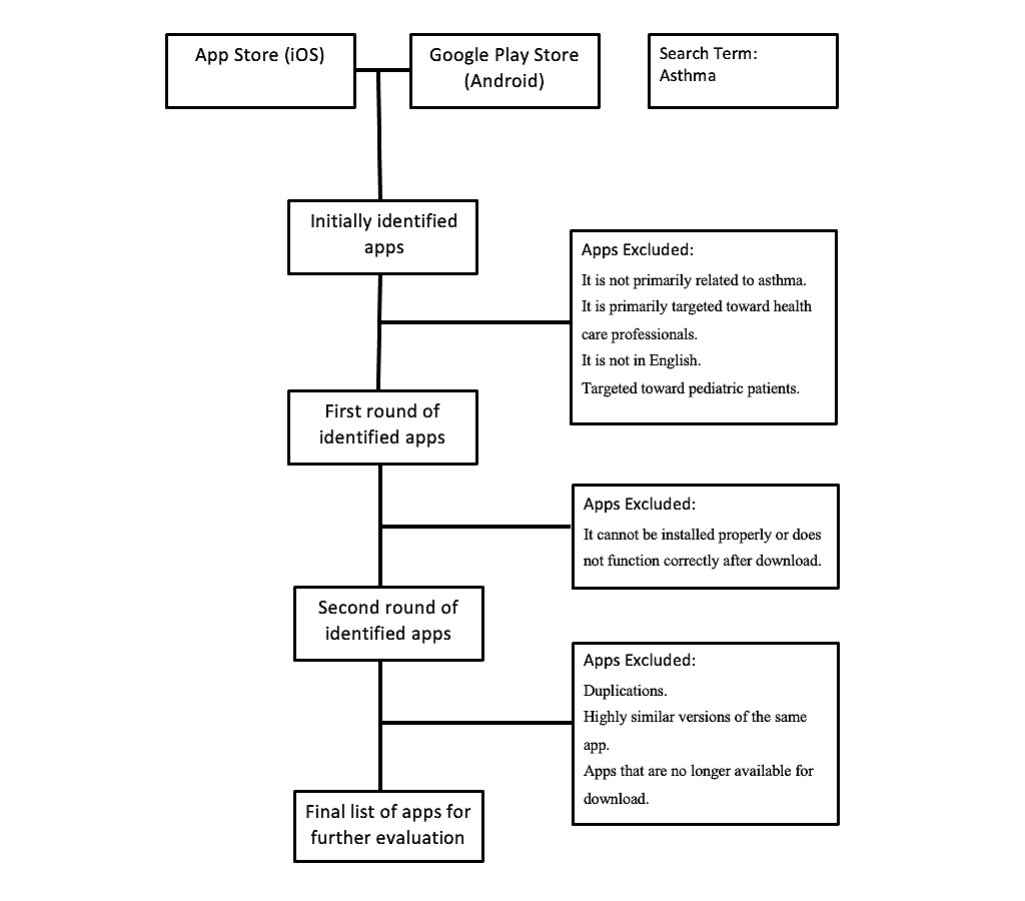
Flowchart of the screening process of apps to arrive at a final list for further evaluation.

After this, we will have a complete list of apps to be reviewed in the evaluation and data extraction. Figure 1 shows a flowchart of the screening process of these apps.

### App Evaluation and Data Extraction

#### Overview

An internet database will be established on Qualtrics (Qualtrics International Inc) to standardize data extraction. This will allow the various researchers to review and enter data from their various places of work. From this software, we will be able to export the information for the analysis of the results later in the study.

As with previous studies that have conducted a similar review of mHealth apps, 2 assessors will review the description of each of the apps selected above, download it, and use the app for a minimum of 20 minutes to be familiar with all functions of the app [11]. There will be a total of 3 app reviewers in this project. Using a web-based team generator, all apps suitable for evaluation will be randomly allocated to assessors so that each app will be appraised by 2 reviewers. The combination of this random allocation and having each app evaluated by 2 reviewers independently will help reduce bias. At the same time, ensuring that each reviewer does not have an inordinate number of apps to assess will serve to prevent overburdening the reviewer with workload to optimize task performance.

The reviewers will subsequently conduct the evaluation and enter the data into the Qualtrics database. Each reviewer will perform this process individually. If, at any stage, a reviewer has a question regarding a certain feature of an app and how to assess it, they will be advised to talk to one of the senior members of the team not involved in the review process.

There are 4 key aspects of the app evaluation and data extraction process. Below are the summaries of these, with the checklists provided in Multimedia Appendix 3 and a step-by-step guide to data collection in Multimedia Appendix 4 [3,12-14].

#### Technical Information About the App

The first step of data collection will involve gathering basic technical information about the app. The decision of which technical information to include is based on prior app review studies [6,11,15]. This will be derived from publicly available information in the in-store app descriptions and any in-app information sections. If required, the app developer’s website will be consulted. Data that will be collected regarding the technical information include the app name, date of release, date of update, developer, developer affiliations, price, rating, number of ratings, platform or platforms, size of the app, and number of downloads. The main purpose of this category is for descriptive purposes when discussing the other findings of the review [15]. This checklist is provided in Multimedia Appendix 3.

#### App Quality Assessment

This assessment will be completed using the validated MARS to objectively determine the quality of the apps selected above [15,16]. This scale has 4 separate domains that are assessed to evaluate mobile app quality. These are engagement, functionality, esthetics, and information quality [15,16]. A total of 19 items, each with a 5-point scale regarding the quality in these 4 domains, makes up the MARS [15]. This framework is presented in Multimedia Appendix 3. Once this has been completed for an app, a mean score for that domain and the overall MARS will be calculated. Following these objective questions, there are subjective questions to evaluate user satisfaction and the perceived impact of the app on the user’s knowledge, attitude, motivation to change, the likelihood of change, and awareness of the importance of changing their asthma self-management [15].

#### App Functionality

App functionality refers to what the app can do for a user and is an important marker of whether an app offers much benefit to users and the overall quality of an app. Although the MARS framework looks at the overall quality of a mobile app, it predominantly focuses on performance, ease of use, gestural design navigation, and navigation of the app [15]. For this reason, the Intercontinental Medical Statistics Institute for Health Informatics Functionality Scoring System, henceforth known as the *IMS functionality score*, will be used. This score has been developed by the above institute and is based on 7 functionality criteria and 4 subcategories in the *record* functionality criterion. This score focuses on the scope of functions, including the ability of the app to inform, instruct, record, display, guide, remind or alert, and communicate information [11,17]. Each app is assessed for having or not having these functions and is then given a total functionality score between 0 and 11 [17]. This scoring system is presented in Multimedia Appendix 3.

#### Presence of App Features Consistent With Asthma Guidelines

A review of the available literature using the CINAHL, MEDLINE, Embase, and PubMed databases was performed to look at the work previous studies conducting a review of asthma apps have done. Although we were able to identify a study that developed a framework for the assessment of asthma smartphone apps, no validated checklists or instruments were identified [10]. In the study, the researchers surveyed the professionals involved to develop an attributed weight of importance for each item using a Delphi survey technique [10]. As discussed above, although derived from parts of the framework by Guan et al [10], our checklist will not be identical to theirs. The main functions of the app that we will be interested in assessing in our checklist include asthma information, self-management skill training (including peak flow use, inhaler technique, and nonpharmacological strategies), monitoring of asthma symptoms, risk evaluation, and prompting (medication reminders and referring to action plan reminders and suggestions for seeking health advice).

The checklist that we developed and that will be used is provided in Multimedia Appendix 3.

Each of the selected apps will be assessed using this checklist, and the data will be extracted into the database discussed above. Where significant differences in judgment are identified, a senior team member not involved in the original review will be alerted and review the app to make the final decision.

### Reviewer Training

A training session will be organized among all the reviewers before the initial data extraction. This training session will be similar to the one performed by Gong et al [11] for their diabetes app review. The goals of this session will be to ensure all participants (1) understand the scope and purpose of the study, (2) understand this study protocol and have read it in full, (3) understand how to search for apps on the Google Play Store and Apple App Store, (4) understand how to extract information from these apps, and (5) understand how to enter data into our web-based database.

This training will be in the form of a web-based lecture; step-by-step examples via screen sharing features; and, finally, case studies, with reviewers expected to use the protocol to assess 5 apps. If the reviewers reach ≥90% agreement, the main study can begin. If the reviewers are <90% in agreement, a further 5 apps will be assessed. If this is an ongoing issue, then the protocol will be examined for flaws and areas of improvement. This session will be conducted in 2 components. In the first session, BR will talk with the other 2 reviewers regarding asthma self-management and how to complete the created checklist. The second session will be run by EG, who has extensive experience in mHealth app reviews and the use of the MARS framework and will instruct reviewers on how to use this appropriately.

A step-by-step reference guide has also been created to inform reviewers on how to fill out the various frameworks and checklists involved in the study. This is provided in Multimedia Appendix 4.

### Quality Assurance, Data Management, and Data Analysis

Quality control is ensured by a number of different methods. First, adequate training will be provided to all the researchers. Second, the selected apps will be allocated to reviewers by web-based random allocation software in an effort to reduce selection bias. Third, 2 reviewers have assessed all mobile apps independently. Finally, discrepancies in opinion between reviewers will be solved by a third senior team member. A total of 2 different major app databases will be used to reduce the risk of selection bias. Given that this is a review of apps and not articles, there are no unpublished or gray literature searches that need to be done, reducing the risk of publication bias.

All data will be entered into either our Microsoft Excel spreadsheet during the screening process or the web-based database when evaluating the apps. These will be stored on a cloud-based system to which only the team has access.

Once ready, the data will be retrieved from these sources, and a descriptive analysis will be performed using data analysis software.

## Results

The above information will be collected in the web-based Qualtrics database and Microsoft Excel spreadsheet. Once completed, all of these data will be downloaded for subsequent analysis. This analysis will comprise a descriptive analysis and calculation of the mean and SD. In the event of skewed data distribution, the IQR and median will be calculated. Given that each app will be double-rated by 2 separate reviewers, interrater reliability scores will be calculated.

At this stage, we plan to conduct all data analyses using Stata statistical software version 14 (StataCorp LLC). We will generate visual figures to demonstrate the results in an easy-to-interpret, visually friendly manner using Microsoft Excel (version 16, Office 365). This study is expected to conclude in late 2022.

## Discussion

### Comparison With Prior Work

This is not the first review of available mHealth asthma apps. Prior studies that have conducted these assessments have primarily focused on evaluating the quality and functionality of apps using the MARS framework, as we do in this study [6-8]. From a review of the literature over the past 5 years, only 2 prior studies were found to have conducted some sort of assessment of the alignment of apps with asthma self-management principles. Both of these studies only looked at free apps, eliminating a number of apps from review [8,18]. The data collection for both reviews occurred >4 years ago [8,18]. In the rapidly developing marketplace of mobile apps, a number of new apps have been released in this time. Our review will look at both free and paid apps and provide an updated assessment, given that our data collection will take place in 2021. Furthermore, Househ et al [18] did not assess apps from the Apple App Store, focusing only on the Google Play Store, and therefore did not fully assess the breadth of available English-language apps in the marketplace. These authors evaluated whether apps included or did not include information consistent with GINA guidelines as per a checklist created by 1 author [18]. However, this was limited to asthma information and education and did not include further features such as the ability of an app to track information, provide asthma skill training, or personalize information. This review also did not examine the overall app quality using the validated MARS framework [18]. Our review benefits from having 2 independent clinicians review the guidelines to establish all GINA self-management recommendations that could be feasibly incorporated into an mHealth app and review app quality using the MARS framework. Furthermore, we will examine not only the presence of information but also the presence or absence of the ability to track asthma symptoms and provide reminders and skill training and all features derived from the GINA guidelines that are provided in Multimedia Appendix 3. As part of their app review, Tan et al [8] established a framework for assessing the alignment of mHealth apps with the theoretical principles of self-management of allergic rhinitis or asthma [8]. A total of 6 asthma self-management principles were identified based on a literature review and author consensus [8]. Our review has taken the further step of specifically deriving the self-management principles from the international best practice GINA guidelines and creating a more extensive checklist looking at these principles. Therefore, the inclusion of paid apps, the creation of an asthma self-management principle checklist derived from international best practice guidelines, and the up-to-date nature of this study will make our study unique.

### Projected Significance

The projected significance of this review is 3-fold. First, it adds to the body of literature on this topic. The systematic approach that we have taken in developing the methodology for this project and the asthma self-management principle checklist will result in a robust evaluation of the quality and content alignment with guidelines of the available mHealth apps. Second, by examining the consistency of these apps with international best practice guidelines, the results will assist clinicians in providing evidence-based advice to adult patients wishing to use mHealth apps as part of their asthma self-management. Finally, by performing this review, we will be able to identify what asthma mHealth apps do well in and what they need improvement in. This will assist in the future development of evidence-based asthma mHealth apps and future research.

### Conclusions

This review represents the first study to our knowledge to examine all English-language mHealth apps available in Australia that are targeted to adults with asthma for their functionality, quality, and consistency with international best practice guidelines. Most apps identified in this review will also be available in other English-speaking regions; thus, the results should be largely generalizable to these regions. The results of this review will help fill gaps in the literature and assist clinicians in providing evidence-based advice to adult patients wishing to use mHealth apps as part of their asthma self-management. Furthermore, it will assist in identifying current gaps in the quality and content of available mHealth apps to develop robust, evidence-based asthma mHealth apps in the future.

## Appendix

Multimedia Appendix 1 Example of a table for recording app store search results.

Multimedia Appendix 2 Table for determining inclusion or exclusion of an app.

Multimedia Appendix 3 Data extraction forms (technical information, Mobile App Rating Scale, Intercontinental Medical Statistics functionality score, and asthma assessment checklist).

Multimedia Appendix 4 Stepwise guide on the key steps of data extraction and evaluation.

## Abbreviations

GINA: Global Initiative for Asthma
MARS: Mobile App Rating Scale
mHealth: mobile health
PRISMA: Preferred Reporting Items for Systematic Reviews and Meta-Analyses
