# Supplementary material for: Functionality and Quality of Asthma mHealth Apps and Their Consistency With International Guidelines: Protocol for a Systematic Search and Evaluation of Mobile Apps
Source: JMIR Res Protoc. 2022 Feb 9;11(2):e33103. doi: 10.2196/33103 (PMC8867297; doi:10.2196/33103)
Supplement: Multimedia Appendix 1 [file resprot_v11i2e33103_app1.docx]

# Appendices

## Appendix One: Example of table for recording App store search results

| **Date of Search** | 05/05/2021 |
| --- | --- |
| **Reviewer** | Billy Robinson |
| **Store Searched** | Apple Store |
| **Search Term** | Asthma |
| **Total Number of Apps Identified** | xxxx |
| **App ID Number (created for this review)** | **App Names** |
| **1.** | Asthma Aust |
| **2.** | Asthma inhaler diary |
| **3.** | Etc. |
