# Supplementary material for: Functionality and Quality of Asthma mHealth Apps and Their Consistency With International Guidelines: Protocol for a Systematic Search and Evaluation of Mobile Apps
Source: JMIR Res Protoc. 2022 Feb 9;11(2):e33103. doi: 10.2196/33103 (PMC8867297; doi:10.2196/33103)
Supplement: Multimedia Appendix 2 [file resprot_v11i2e33103_app2.docx]

## Appendix Two: Table for determining inclusion/exclusion of an App

|  |  |  |  |  |  |  |  |  |  |  |
| --- | --- | --- | --- | --- | --- | --- | --- | --- | --- | --- |

| Reviewer Name : XX  Review Start Date: XX XX XXXX  Review End Date: XX XX XXXX | | | | | | | | | | | |  |
| --- | --- | --- | --- | --- | --- | --- | --- | --- | --- | --- | --- | --- |
| **Application Information** | | **Inclusion Criteria** | | | | **Exclusion Criteria** | | | | **Decision** | |  |
| App Number | Application Name | Primary role related to asthma | Targeted to those with asthma | It can be run on mobile phones | Written in English | Not primarily related to asthma | Primarily targeted towards healthcare professionals | Not written in English | Designed for children with asthma | Included? (Y/N) | Reason for Exclusion (if applicable) | |
| 1 | Asthma Aust | Y | Y | Y | Y | N | N | N | N | Y |  | |
| 2 | FakeAsthma App | Y | N | N | Y | N | Y | N | N | N | Primarily targeted towards healthcare professionals | |
|  |  |  |  |  |  |  |  |  |  |  |  | |
|  |  |  |  |  |  |  |  |  |  |  |  | |
|  |  |  |  |  |  |  |  |  |  |  |  | |
|  |  |  |  |  |  |  |  |  |  |  |  | |
|  |  |  |  |  |  |  |  |  |  |  |  | |
