# Supplementary material for: Functionality and Quality of Asthma mHealth Apps and Their Consistency With International Guidelines: Protocol for a Systematic Search and Evaluation of Mobile Apps
Source: JMIR Res Protoc. 2022 Feb 9;11(2):e33103. doi: 10.2196/33103 (PMC8867297; doi:10.2196/33103)
Supplement: Multimedia Appendix 3 [file resprot_v11i2e33103_app3.docx]

## Appendix Three: Data Extraction Forms (Technical information, MARS, IMS Functionality score and Asthma Assessment Checklist). *N.b. these will be transcribed into the online Qualtrics survey software.*

Name of reviewer: ___________________ Date of review: ______________________

How long have you tried this app before the rating? _____day_____h ______minutes

App store: ☐ Apple store ☐Google Play

### Section I. Basic information (adjusted based on App Classification part of MARS scale):

*Note: most of the information in this section could be found through App description in App store.*

a1. **Full name of the APP:** __________________________

a2. **Version of the APP:** ___________________

a3. **Date of last update:** ☐☐☐☐/☐☐/☐☐ (yyyy/mm/dd)

a4. **Size of the App**: _____________(Mb)

a5**. Full name of the Developer:** _____________

a6. **Developer Affiliation**: ☐technical company ☐healthcare/pharmaceutical company ☐clinical institution ☐ research institution ☐ NGO ☐ government ☐ other(specify) ☐ unknown

a7. **Language (all that apply):** ☐ English ☐others: ____________

a8. **Number of ratings**：__________________

a9. **Rating score:** _____________________

a10. **Number of download (if available):** ________________

a11. **Cost for basic or stand-alone version:** ☐Free, ☐Not free, ______ AUD

a12. **Cost for upgrade version**: ☐Free ☐Not Applicable (there is no upgrade version) ☐Not free, ______ AUD

a13. **Technical aspects of app (all that apply)**

☐Allows sharing (Facebook, Twitter etc) ☐Has an app community

☐Allows password-protection ☐Requires login

☐Sends reminders ☐Needs web access to function

a14. **Theoretical background/Strategies (all that apply)**

☐Assessment ☐Feedback ☐Information/Education ☐Monitoring/Tracking ☐Goal setting

☐Advice /Tips /Strategies /Skills training

☐CBT - Behavioral (positive events) ☐CBT –Cognitive (thought challenging)

☐ACT - Acceptance commitment therapy

☐Mindfulness/Meditation ☐Relaxation ☐Gratitude ☐Strengths based ☐Other ____________________________

### Section II. App Quality Ratings (Mobile App Rating Scale)

1. **Engagement – fun, interesting, customizable, interactive (e.g. sends alerts, messages, reminders, feedback, enables sharing), well-targeted to audience**

1. Entertainment: Is the app fun/entertaining to use? Does it use any strategies to increase engagement through entertainment (e.g. through gamification)?

- 1 Dull, not fun or entertaining at all
- 2 Mostly boring
- 3 OK, fun enough to entertain user for a brief time (< 5 minutes)
- 4 Moderately fun and entertaining, would entertain user for some time (5-10 minutes total)
- 5 Highly entertaining and fun, would stimulate repeat use

Note: (write done if it is not relevant, applicable, or the decision is hard to be made.)________________________________________________________________________

2. Interest: Is the app interesting to use? Does it use any strategies to increase engagement by presenting its content in an interesting way?

- 1 Not interesting at all
- 2 Mostly uninteresting
- 3 OK, neither interesting nor uninteresting; would engage user for a brief time (< 5 minutes)
- 4 Moderately interesting; would engage user for some time (5-10 minutes total)
- 5 Very interesting, would engage user in repeat use

Note: _________________________________________________________________________

3. Customization: Does it provide/retain all necessary settings/preferences for apps features (e.g.

sound, content, notifications, etc.)?

- 1 Does not allow any customization or requires setting to be input every time
- 2 Allows insufficient customization limiting functions
- 3 Allows basic customization to function adequately
- 4 Allows numerous options for customization
- 5 Allows complete tailoring to the individual’s characteristics/preferences, retains all settings

Note: _________________________________________________________________________

4. Interactivity: Does it allow user input, provide feedback, contain prompts (reminders, sharing

options, notifications, etc.)? Note: these functions need to be customizable and not

overwhelming in order to be perfect.

- 1 No interactive features and/or no response to user interaction
- 2 Insufficient interactivities, or feedback, or user input options, limiting functions
- 3 Basic interactive features to function adequately
- 4 Offers a variety of interactive features/feedback/user input options
- 5 Very high level of responsiveness through interactive features/feedback/user input options

Note: _________________________________________________________________________

5. Target group: Is the app content (visual information, language, design) appropriate for your

target audience?

- 1 Completely inappropriate/unclear/confusing
- 2 Mostly inappropriate/unclear/confusing
- 3 Acceptable but not targeted. May be inappropriate/unclear/confusing
- 4 Well-targeted, with negligible issues
- 5 Perfectly targeted, no issues found

Note: _________________________________________________________________________

1. **Functionality – app functioning, easy to learn, navigation, flow logic, and gestural design of app**

6. Performance: How accurately/fast do the app features (functions) and components

(buttons/menus) work?

- 1 App is broken; no/insufficient/inaccurate response (e.g. crashes/bugs/broken features, etc.)
- 2 Some functions work, but lagging or contains major technical problems
- 3 App works overall. Some technical problems need fixing/Slow at times
- 4 Mostly functional with minor/negligible problems
- 5 Perfect/timely response; no technical bugs found/contains a ‘loading time left’ indicator

Note: _________________________________________________________________________

7. Ease of use: How easy is it to learn how to use the app; how clear are the menu labels/icons and instructions?

- 1 No/limited instructions; menu labels/icons are confusing; complicated
- 2 Useable after a lot of time/effort
- 3 Useable after some time/effort
- 4 Easy to learn how to use the app (or has clear instructions)
- 5 Able to use app immediately; intuitive; simple

Note: _________________________________________________________________________

8. Navigation: Is moving between screens logical/accurate/appropriate/ uninterrupted; are all

necessary screen links present?

- 1 Different sections within the app seem logically disconnected and random/confusing/navigation is difficult
- 2 Usable after a lot of time/effort
- 3 Usable after some time/effort
- 4 Easy to use or missing a negligible link
- 5 Perfectly logical, easy, clear and intuitive screen flows throughout, or offers shortcuts

Note: _________________________________________________________________________

9. Gestural design: Are interactions (taps/swipes/pinches/scrolls) consistent and intuitive across

all components/screens?

- 1 Completely inconsistent/confusing
- 2 Often inconsistent/confusing
- 3 OK with some inconsistencies/confusing elements
- 4 Mostly consistent/intuitive with negligible problems
- 5 Perfectly consistent and intuitive

Note: _________________________________________________________________________

**C****.** **Aesthetics – graphic design, overall visual appeal, color scheme, and stylistic consistency**

10. Layout: Is arrangement and size of buttons/icons/menus/content on the screen appropriate or zoomable if needed?

- 1 Very bad design, cluttered, some options impossible to select/locate/see/read device display not optimized
- 2 Bad design, random, unclear, some options difficult to select/locate/see/read
- 3 Satisfactory, few problems with selecting/locating/seeing/reading items or with minor screen size problems
- 4 Mostly clear, able to select/locate/see/read items
- 5 Professional, simple, clear, orderly, logically organized, device display optimized. Every design component has a purpose

Note: _________________________________________________________________________

11. Graphics: How high is the quality/resolution of graphics used for buttons/icons/menus/content?

- 1 Graphics appear amateur, very poor visual design - disproportionate, completely stylistically inconsistent
- 2 Low quality/low resolution graphics; low quality visual design – disproportionate, stylistically inconsistent
- 3 Moderate quality graphics and visual design (generally consistent in style)
- 4 High quality/resolution graphics and visual design – mostly proportionate, stylistically consistent
- 5 Very high quality/resolution graphics and visual design - proportionate, stylistically consistent throughout

Note: _________________________________________________________________________

12. Visual appeal: How good does the app look?

- 1 No visual appeal, unpleasant to look at, poorly designed, clashing/mismatched colours
- 2 Little visual appeal – poorly designed, bad use of colour, visually boring
- 3 Some visual appeal – average, neither pleasant, nor unpleasant
- 4 High level of visual appeal – seamless graphics – consistent and professionally designed
- 5 As above + very attractive, memorable, stands out; use of colour enhances app features/menus

Note: _________________________________________________________________________

1. **Information – Contains high quality information (e.g. text, feedback, measures, references) from a credible source. Select N/A if the app component is irrelevant.**

13. Accuracy of app description (in app store): Does app contain what is described?

- 1 Misleading. App does not contain the described components/functions. Or has no description
- 2 Inaccurate. App contains very few of the described components/functions
- 3 OK. App contains some of the described components/functions
- 4 Accurate. App contains most of the described components/functions
- 5 Highly accurate description of the app components/functions

Note: _________________________________________________________________________

14. Goals: Does app have specific, measurable and achievable goals (specified in app store

description or within the app itself)?

- N/A Description does not list goals, or app goals are irrelevant to research goal (e.g. using a game for educational purposes)
- 1 App has no chance of achieving its stated goals
- 2 Description lists some goals, but app has very little chance of achieving them
- 3 OK. App has clear goals, which may be achievable.
- 4 App has clearly specified goals, which are measurable and achievable
- 5 App has specific and measurable goals, which are highly likely to be achieved

Note: _________________________________________________________________________

15. Quality of information: Is app content correct, well written, and relevant to the goal/topic of the app?

- N/A There is no information within the app
- 1 Irrelevant/inappropriate/incoherent/incorrect
- 2 Poor. Barely relevant/appropriate/coherent/may be incorrect
- 3 Moderately relevant/appropriate/coherent/and appears correct
- 4 Relevant/appropriate/coherent/correct
- 5 Highly relevant, appropriate, coherent, and correct

Note: _________________________________________________________________________

16. Quantity of information: Is the extent coverage within the scope of the app; and comprehensive but concise?

- N/A There is no information within the app
- 1 Minimal or overwhelming
- 2 Insufficient or possibly overwhelming
- 3 OK but not comprehensive or concise
- 4 Offers a broad range of information, has some gaps or unnecessary detail; or has no links to more information and resources
- 5 Comprehensive and concise; contains links to more information and resources

Note: _________________________________________________________________________

17. Visual information: Is visual explanation of concepts – through charts/graphs/images/videos, etc. – clear, logical, correct?

N/A There is no visual information within the app (e.g. it only contains audio, or text)

- 1 Completely unclear/confusing/wrong or necessary but missing
- 2 Mostly unclear/confusing/wrong
- 3 OK but often unclear/confusing/wrong
- 4 Mostly clear/logical/correct with negligible issues
- 5 Perfectly clear/logical/correct

Note: _________________________________________________________________________

18. Credibility: Does the app come from a legitimate source (specified in app store description or

within the app itself)?

- 1 Source identified but legitimacy/trustworthiness of source is questionable (e.g. commercial business with vested interest)
- 2 Appears to come from a legitimate source, but it cannot be verified (e.g. has no webpage)
- 3 Developed by small NGO/institution (hospital/center, etc.) /specialized commercial business, funding body
- 4 Developed by government, university or as above but larger in scale
- 5 Developed using nationally competitive government or research funding (e.g. Australian Research Council, NHMRC)

Note: _________________________________________________________________________

19. Evidence base: Has the app been trialed/tested; must be verified by evidence (in published

scientific literature)?

- N/A The app has not been trialed/tested
- 1 The evidence suggests the app does not work
- 2 App has been trialled (e.g., acceptability, usability, satisfaction ratings) and has partially positive outcomes in studies that are not randomized controlled trials (RCTs), or there is little or no contradictory evidence.
- 3 App has been trialled (e.g., acceptability, usability, satisfaction ratings) and has positive
- outcomes in studies that are not RCTs, and there is no contradictory evidence.
- 4 App has been trialled and outcome tested in 1-2 RCTs indicating positive results
- 5 App has been trialled and outcome tested in > 3 high quality RCTs indicating positive results

Note: _________________________________________________________________________

**E. subjective quality**

20. Would you recommend this app to people who might benefit from it?

- 1 Not at all I would not recommend this app to anyone
- 2 There are very few people I would recommend this app to
- 3 Maybe There are several people whom I would recommend it to
- 4 There are many people I would recommend this app to
- 5 Definitely I would recommend this app to everyone

21. How many times do you think you would use this app in the next 12 months if it was

relevant to you?

- 1 None
- 2 1-2
- 3 3-10
- 4 10-50
- 5 >50

22. Would you pay for this app?

- 1 No
- 3 Maybe
- 5 Yes

23. What is your overall star rating of the app?

- 1 one star, one of the worst apps I’ve used
- 2 two stars
- 3 three stars, Average
- 4 four stars
- 5 five stars, one of the best apps I've used

Note:_________________________________________________________________________

**Section F: App-specific**

These added items can be adjusted and used to assess the perceived impact of the app on the user’s knowledge, attitudes, intentions to change as well as the likelihood of actual change in the target health behaviour.

|  | **Strongly disagree** |  |  |  | **Strongly agree** |
| --- | --- | --- | --- | --- | --- |
|  | 1 | 2 | 3 | 4 | 5 |
| Awareness: This app is likely to increase awareness of the importance of addressing asthma self-management |  |  |  |  |  |
| Knowledge: This app is likely to increase knowledge/understanding of asthma self-management |  |  |  |  |  |
| Attitudes: This app is likely to change attitudes toward improving asthma self-management |  |  |  |  |  |
| Intention to change: This app is likely to increase intentions/motivation to address asthma |  |  |  |  |  |
| Help seeking: Use of this app is likely to encourage further help seeking for asthma management |  |  |  |  |  |
| Behaviour change: Use of this app is likely decrease asthma exacerbations |  |  |  |  |  |

## Section III. IMS institute for Healthcare Informatics functionality scoring criteria

One point for each of the following. Apps will have a rating between 0-11.

- **Inform:** Provides information in a variety of formats (text, photo, video)
- **Instruct:** Provides instructions to the user
- **Record:** Capture user entered data
- Collect data: Able to enter and store health data on individual phone
- Share data: Able to transmit health data
- Evaluate data: Able to evaluate the entered health data by patient and provider, provider and administrator, or patient and caregiver
- Intervene: Able to send alerts based on the data collected or propose behavioural intervention or changes
- **Display:** Graphically display user entered data/output user entered data
- **Guide:** Provide guidance based on user entered information, and may further offer a diagnosis, or recommend a consultation with a physician/a course of treatment
- **Remind or Alert** Provide reminders to the user
- **Communicate** Provide communication with healthcare provider/patients and/or provide links to social networks

## Section IV. Presence of App Features consistent with Asthma Guidelines

4. 1 Asthma Education/Knowledge

|  | **Provide knowledge on** | | **The knowledge is individualized** | | **The knowledge is evidence-based** | | **Breadth of Knowledge provided?**  **<50%=Poor**  **50-80%=Good**  **80-100%=Exceptional** | | |
| --- | --- | --- | --- | --- | --- | --- | --- | --- | --- |
|  | **Yes** | **No** | **Yes** | **No** | **Yes** | **No** | **Poor** | **Good** | **Exceptional** |
|  |  |  |  |  |  |  |  |  |  |
| **General knowledge about Basic Facts of Asthma** |  |  |  |  |  |  |  |  |  |
| Definition of Asthma |  |  |  |  |  |  |  |  |  |
| Causes of asthma |  |  |  |  |  |  |  |  |  |
| Symptoms of Asthma |  |  |  |  |  |  |  |  |  |
| Prognosis of asthma |  |  |  |  |  |  |  |  |  |
| Early treatment of asthma |  |  |  |  |  |  |  |  |  |
| **Asthma Medications** |  |  |  |  |  |  |  |  |  |
| Categories of Asthma medications |  |  |  |  |  |  |  |  |  |
| Roles and usage of controller medications |  |  |  |  |  |  |  |  |  |
| Roles and usage of reliver medications |  |  |  |  |  |  |  |  |  |
| Importance of adherence to preventer medication |  |  |  |  |  |  |  |  |  |
| Importance of not only using SABA therapy |  |  |  |  |  |  |  |  |  |
| Medication side effects |  |  |  |  |  |  |  |  |  |
| Importance of carrying reliever medications |  |  |  |  |  |  |  |  |  |
| Importance of using a spacer |  |  |  |  |  |  |  |  |  |
| **Asthma exacerbation management** |  |  |  |  |  |  |  |  |  |
| Early signs and symptoms of worsening asthma |  |  |  |  |  |  |  |  |  |
| Symptoms of asthma flare |  |  |  |  |  |  |  |  |  |
| Management of asthma exacerbations |  |  |  |  |  |  |  |  |  |
| Management after exacerbation |  |  |  |  |  |  |  |  |  |
| **Asthma risk factors and triggers** |  |  |  |  |  |  |  |  |  |
| Modifiable risk factors for asthma |  |  |  |  |  |  |  |  |  |
| Advises where to seek further help managing these risk factors |  |  |  |  |  |  |  |  |  |
| Triggers for asthma |  |  |  |  |  |  |  |  |  |

4. 2 Provides skill training for asthma self-management

|  | **Provides general skill training** | | **Provides personalised skills training** | |
| --- | --- | --- | --- | --- |
|  | **Yes** | **No** | **Yes** | **No** |
|  |  |  |  |  |
| **Peak Flow Meter Use** |  |  |  |  |
| Describes why and when to use peak flow meter |  |  |  |  |
| Describes Operational Criteria for Peak Flow Meter |  |  |  |  |
| Demonstrates through photos/videos peak flow use |  |  |  |  |
| **Inhaler device use** |  |  |  |  |
| Describes how to use a spacer |  |  |  |  |
| Demonstrates how to use a spacer through videos/photos |  |  |  |  |
| Demonstrates how to care for a spacer |  |  |  |  |
| Describes how to use common inhaler devices |  |  |  |  |
| Demonstrates how to use common inhaler devices through videos/photos |  |  |  |  |
| **Recognise and Respond to Exacerbations** |  |  |  |  |
| Encourages patients to monitor for signs of asthma exacerbation |  |  |  |  |
| Provide an area for asthma action plan |  |  |  |  |
| Specifically guides patients to use their asthma action plan |  |  |  |  |
| Provide information on how to use an asthma action plan |  |  |  |  |
| Prompts patient to see health care provider when required |  |  |  |  |
| **Non-Pharmacological Management Strategies Reduce Asthma Exacerbations** |  |  |  |  |
| Helps identify triggers that make symptoms worse |  |  |  |  |
| Advises avoidance of environmental smoke exposure |  |  |  |  |
| Advises avoidance of medications that can worsen asthma |  |  |  |  |
| Advises avoidance of occupation exposures |  |  |  |  |
| Advises on the avoidance of allergen exposure |  |  |  |  |
| Advises on avoidance of indoor/outdoor pollution |  |  |  |  |
| Advises on avoidance of emotional stress |  |  |  |  |
| Advises on regular moderate physical activity |  |  |  |  |

- 1. Tracking and displaying health information

|  | **The App could support tracking and recording data on** | | **What approach is the data collected?** | | | **Are there tables/graphs displaying trends/analysis results?** | | | **Can this information be easily exported i.e. through email to healthcare providers?** | | **These features are able to be individualized** | |
| --- | --- | --- | --- | --- | --- | --- | --- | --- | --- | --- | --- | --- |
|  | **Yes** | **No** | **Manual input** | **Transfer through external sensors**  **/devices** | **Other** | **Yes, summary without data analysis** | **Yes, summary with data analysis** | **No** | **Yes** | **No** | **Yes** | **No** |
|  |  |  |  |  |  |  |  |  |  |  |  |  |
| **Asthma Symptoms** |  |  |  |  |  |  |  |  |  |  |  |  |
| **Night Waking Due to Asthma** |  |  |  |  |  |  |  |  |  |  |  |  |
| **Activity Limitation due to Asthma** |  |  |  |  |  |  |  |  |  |  |  |  |
| **Peak Flow Meter Values** |  |  |  |  |  |  |  |  |  |  |  |  |
| **SABA Use** |  |  |  |  |  |  |  |  |  |  |  |  |
| **Preventer adherence** |  |  |  |  |  |  |  |  |  |  |  |  |

4.4 The App Provides Prompts on:

|  | **Reminder on** | | **The reminders are individualized** | |
| --- | --- | --- | --- | --- |
|  | **Yes** | **No** | **Yes** | **No** |
|  |  |  |  |  |
| Assessing asthma symptoms over the last month |  |  |  |  |
| Appointment with physicians |  |  |  |  |
| Performing peak flow test |  |  |  |  |
| Preventer medication adherence |  |  |  |  |
| Checking the date of expiry and dosage of inhalers |  |  |  |  |
| Warning of changing health data (i.e. very frequent exacerbations) |  |  |  |  |
| Seeking urgent health advice based on the health data the user inputs into the App |  |  |  |  |

**4.5. The users could make appointment with physicians through the App?**

**☐** Yes, online consultation ☐ Yes, face-to-face consultation ☐ No

**4.6. The App provides an area for patients to keep record of their Asthma Action Plan**

**☐** Yes, can type in action plan ☐ Yes, upload a photo of action plan for easy access ☐ No

**4.7 Communication-forum: The App includes social forums/blogs that promote peer-support and communication among asthma patients.** ☐ Yes ☐ No

**4.8. Communication-record sharing: The users could send the recorded data to others ☐ Yes, to physicians** ☐ Yes, to other family members ☐No

**4.9. The App could help the users to evaluate the risk of having future asthma exacerbations** ☐ Yes using a validated scoring system, ☐ Yes, but not using a validated scoring system ☐ No

**4.10 . The App could guide the users to find out the closest pharmacy store/clinic/hospital?** ☐ Yes ☐ No

**4.11. The App uses recognised screening, symptom control and/or numerical asthma control tools ☐** Yes, all three **☐** Yes, screening tool ☐ Yes, symptom control tool ☐ Yes numerical asthma control tools ☐ No

**4.12. The App allows users to connect to wearables technology. Select all that apply. ☐** Yes **☐** Not an available feature. ☐ Smart Watch ☐ Activity sensor (Fitbit etc) ☐ Smart Peak Flow Meter ☐ Handheld Spirometer ☐ SpO2 Sensor ☐ Other. Specify ___________________

**4.13 Any other functions that are not listed above?** ☐ Yes, specify___________ ☐ No
