# Supplementary material for: Functionality and Quality of Asthma mHealth Apps and Their Consistency With International Guidelines: Protocol for a Systematic Search and Evaluation of Mobile Apps
Source: JMIR Res Protoc. 2022 Feb 9;11(2):e33103. doi: 10.2196/33103 (PMC8867297; doi:10.2196/33103)
Supplement: Multimedia Appendix 4 [file resprot_v11i2e33103_app4.docx]

## Appendix Four: Stepwise guide on the key steps of data extraction and evaluation

### Step 1. Things you need to know before doing any app review

Prior to commencing a review of the mobile health applications, you should have a basic understanding of project protocol, asthma clinical features and self-management strategies, behaviour change theories and the tools used in this project. This will be attained by reviewing the key articles sent to you, reviewing the protocol and through the online training sessions. Please read the articles carefully and make sure that you understand and could answer the following questions:

- 1. What is the purpose of this project?
     1. See this protocol
  2. How people with asthma could self-manage their conditions?
     1. See article sent to you from the European Respiratory Society (1) and Appendix 4.4.
  3. What are the clinical features of asthma that a patient should be aware of?
     1. See article sent to you from the Lancet (2) and Chapters 1, 2 and 4 of the 2021 GINA Guidelines. (3)
  4. What are strategies and theories for behaviour change?
     1. See article sent to you by E Gong from the Annals of Behavioural medicine (4)
  5. What are the strategies that you could use to ensure that your rating is objective?
     1. Be conscious of your own biases when answering questions. This will be touched on during our training session.
  6. What should I know if I would like to change an answer in Qualtrics?
     1. This will be discussed during our training session.

Once you are confident with the answering the above questions, and you have completed the training, proceed with application reviews.

### Step 2. Download several applications and try each aspect of the applications.

Time must be devoted to learning all relevant functions and key aspects of the applications. For some functions (such as message reminders) you may need to allow days to fully check how each function works. Reviewing and trying several apps (10-20 apps) before rating any of the app will be very helpful for you to distinguish a suboptimal from excellent presentation. The specific steps for trying the functions of applications is as follows:

1. Search the app name and download the app from the app store.
   1. Read the description of the app in the app store very carefully as there will be data that needs to be extracted into our checklist.
   2. Keep receipts of all paid apps/subscriptions for claiming through the Northern Hospital respiratory department.
      1. In the case that something is subscription based i.e. charged weekly/monthly etc., ensure you unsubscribe from this service once you have reviewed the app.
      2. Mobile phone, internet or cellular data costs will not be reimbursed. If this a concern, please alert the project lead ASAP.
2. You may need to set up accounts to use the mobile health applications. This is not uncommon for mobile applications. Often a Facebook account or Gmail account can be used to sign in.
   1. If you are not comfortable using Facebook for these application sign ins, please create a new Gmail account to use for the project.
   2. In using these Apps you are playing the role of a person with asthma. If there is an option for selecting health conditions, select ‘Asthma’ and nothing else. Given all reviewers are over the age of 18, asthma management principles should be similar for all adults. Select your true age and choose the gender that you identify as. For consistency, if asked, you have no other medical history. If there are other parameters that the application requires on setting up please contact the project lead, Billy Robinson. He will direct you what to put in so that we have consistency on these accounts.
3. Once you have access to an application, each function must be tried.
   1. Click on all tabs, buttons and options of the application to fully explore it.
   2. Data will often need to be entered into the application to see the response provided by the app. Some examples of what to input to improve consistency for common questions/data parameters is:
      1. Regular Inhalers: Twice Daily Budesonide-Formoterol (Symbicort)
      2. Reliever Inhaler frequency
         1. As needed Budesonide-Formoterol (or as needed Salbutamol if this is not an option)
         2. Trial as used once, twice and four times a day
      3. Peak expiratory flow (PEF) readings (trial these two to determine what the app does with a good (1) and poor (2) PEF
         1. PEF >80% of best
         2. PEF 40% of best
      4. Asthma symptoms
         1. Wheeze
         2. Cough
         3. Shortness of breath
         4. Chest tightness
      5. Night waking due to asthma
         1. Trial yes and no to see what the app does
      6. Activity limitation
         1. Trial none, some (can’t exercise as well) and significant (can’t complete ADLs) to see what the app does.
      7. Asthma Action Plan
         1. Some apps will ask you to take a photo of an asthma action plan (just photo a blank action plan from the National Asthma Council Australian) as a reference guide for patients. Others will ask for you to type it into the application. If this is the case, please input the following:
            1. **When Well:**

**Meaning:** Not awakening at night/morning with asthma, not interfering with ADLs.

**Preventer:** Budesonide-Formoterol (Symbicort) 200/6microg. One puff Twice a day. With spacer

**Reliever:** Budesonide-Formoterol (Symbicort) 200/6microg. Two separate puffs (one at a time) as needed. With spacer

- - - - 1. **When not well**

**Meaning:** Over a period of 2-3 days asthma symptoms are getting worse/not improving, using more than 6puffs of reliever Symbicort/day, Peak Flow <60%.

**Preventer:** Budesonide-Formoterol (Symbicort) 200/6microg. One puff Twice a day. With spacer

**Reliever:** Budesonide-Formoterol (Symbicort) 200/6microg. One puff as needed. Up to a maximum of 12 total puffs/day. With spacer

**Steroid:** Start oral Prednisolone 50mg/day for 5-days

**Seek** same day medical attention if >12 puffs of Symbicort/day.

- - - - 1. **If symptoms get worse:**

**Meaning:** Severe asthma flare-up/attack (needing reliever again within 3 hours, increasing difficulty breathing, waking often at night with asthma symptoms)

**Seek** same day medical review

**Preventer:** Budesonide-Formoterol (Symbicort) 200/6microg. One puff Twice a day. With spacer

**Reliever:** Budesonide-Formoterol (Symbicort) 200/6microg. Two separate puffs (one at a time) as needed. Up to a maximum of 12 total puffs/day. With spacer

**Steroid:** Start oral Prednisolone 50mg/day for 5-days

- - - - 1. **Asthma emergency**

**Meaning:** Severe breathing problems, symptoms get worse very quickly, difficulty speaking, reliever has little or no effect

**Contact an ambulance via 000.**

**Remain upright and calm**

**Reliever:** Budesonide-Formoterol (Symbicort) 200/6microg. Two separate puffs (one at a time) as needed. Up to a maximum of 12 total puffs/day. With spacer

### Step 3. After trialing more than 10 applications and you are confident in your ability to explore applications and extract information of interest, start extracting data and doing evaluation in Qualtrics. Note:

1. All information contained in the app must be read. Press/select as many buttons/options as possible. You must trial each feature of the application prior to rating.
2. You must trial the application for 20-30minutes prior to rating.
3. Be aware of personal biases based on personal opinion of the application. Score each question objectively based on the checklist criteria and what is/is not available in the App and how it is presented.
4. Little and often! Do not perform too much rating on a single day. This is a laborious process and there is no rush. When first commencing reviews, you may want to only do one App review/day but this may steadily increase as you become more familiar with the tools. As a general rule of thumb do not rate more than 5 apps per day. The reasons for this are personal and profession. We do not want you to become burnt-out or overworked from the project, particularly as we also have to perform our regular daily jobs and study. Secondly burn-out from too much rating in one day can introduce subjectivity in ratings. Reviewers may start getting confused by features across apps and become personally biased towards features they like and dislike, rather than their availability and implementation, which is what the MARS aims to rate.
5. A WhatsApp group for reviewers will be created. Any quick questions regarding using Qualtrics or what information to put into the application that isn’t included in Step 2, can be sent here and guidance can be provided. If you have questions about uncertainty on what to rate a particular feature of an App please contact one of the project members not involved in reviewing applications for clarification. Reviewers are not to have contact regarding their individual ratings of an application and as a rule interaction between reviewers should be limited. This is to minimize bias.

### Step 4. Question by question guideline on data extraction forms

Example of table for recording App store search results

| **Date of Search** | 05/05/2021 |
| --- | --- |
| **Reviewer** | Billy Robinson |
| **Store Searched** | Apple Store |
| **Search Term** | Asthma |
| **Total Number of Apps Identified** | xxxx |
| **App ID Number (created for this review)** | **App Names** |
| **1.** | Asthma Aust |
| **2.** | Asthma inhaler diary |
| **3.** | Etc. |

### Section I. Basic Information (adjusted based on App Classification part of MARS scale)

|  | Explanation about the questions |
| --- | --- |
| **Basic Information** | *Most of the information in this section could be found through App description in the App stores.* |
| a1. Full name of the APP: __________________________  a2. Version of the APP: ___________________  a3. Date of last update: ☐☐☐☐/☐☐/☐☐ (yyyy/mm/dd)  a4. Size of the App: _____________(Mb) | For a3 date of last update, please fill in based on the format as yyyy/mm/dd |
| a5. Full name of the Developer: _____________  a6. Developer Affiliation: ☐technical company ☐healthcare/pharmaceutical company ☐clinical institution ☐ research institution ☐ NGO ☐ government ☐ other(specify) ☐ unknown | To understand the developer affiliation, you may need to do some further research about the developer online. |
| a7. Language (all that apply): ☐ English ☐others: __________________________________________________________ |  |
| a8. Number of ratings：__________________  a9. Rating score: _____________________ |  |
| a10. Number of download (if available): ________________ |  |
| a11. Cost for basic version: ☐Free ☐Not Applicable (there is no upgrade version) ☐Not free, ______ RMB  a12. Cost for upgrade version: ☐Free ☐Not Applicable (there is no upgrade version) ☐Not free, ______ RMB | Some application may have basic version and upgrade version. Choose free if there is no cost, choose NA if there is no upgrade version. If there is cost for using the upgrade version, please specify the cost. Money spent on purchasing services/products won’t be accounted. |
| a13. Technical aspects of app (all that apply)  ☐Allows sharing (Facebook, twitter, etc.)  ☐Has an app community  ☐Allows password-protection  ☐Requires login  ☐Sends reminders  ☐Needs web access to function | This is a checklist question. Choose all that apply.   - Allow sharing: whether users could share some information through the app to personal network platform such as Facebook, Twitter etc. - Has an app community: means whether the app has the function that App users could communicate within the App - Allows password protection: whether you could set a password to protect the data being seen by others - Requires login: whether you have to register and log in before using the app - Send reminders: whether the app could send reminders on medication taking, reviewing symptoms, physical activities. You may need to try the app for more than a day to capture this function. - Needs web access to function: whether you have to have access to a WiFi or cellular data network to access to certain functions |
| a14. Theoretical background/Strategies (all that apply)  ☐Assessment ☐Feedback ☐Information/Education ☐Monitoring/Tracking ☐Goal setting  ☐Advice /Tips /Strategies /Skills training  ☐CBT - Behavioural (positive events) ☐CBT –Cognitive (thought challenging)  ☐ACT - Acceptance commitment therapy  ☐Mindfulness/Meditation ☐Relaxation ☐Gratitude ☐Strengths based  ☐Other ____________________________ | This question tries to capture the strategies that the app used to help people and promote behaviour changes. Please review the articles sent to you by E Gong for further clarification:   - Assessment: for example, whether the app could assess the risk of a future asthma exacerbation - Feedback: whether the app could provide some feedback to you when you enter the data - Information and education: whether the app contains education to teach users about asthma and asthma management - Monitoring/tracking: whether the app could monitor/track asthma symptoms, inhaler use, physical activity level, peak flow monitor level etc. - Goal setting: For example, whether the app could support users in setting a lifestyle change goal or a preventer compliance goal. - Advice/Tips/Strategies/Skills training: for example, whether the App provides certain tips on how people could improve their inhaler technique, using asthma action plan etc. - CBT - Behavioural (positive events) - CBT –Cognitive (thought challenging) - ACT - Acceptance commitment therapy - Mindfulness/Meditation - Relaxation - Gratitude - Strengths based |

### Section II. APP Quality Rating (Mobile App Rating Scale)

| **Mobile Application Rating Scale** | **Explanation about the questions** |
| --- | --- |
| **Objective Rating Scales** | Objective rating scales contains 19 questions. All questions will be judged by five-point rating scale:   - 1-2 means **inadequate** and **poor**: there must be some important components missing. - 3 means **acceptable**: the app you rate are at common ground level without any big problem - 4-5 means **good** and **excellent**: Some extra efforts have been done by the developer to promote the use of the App - Under each choice, you could add some notes about your concerns if there is any. - **Please make sure that all the rating is objective. You should not add any personal preference when doing the rating.** |
| 1. **Engagement – fun, interesting, customizable, interactive (e.g. sends alerts, messages, reminders, feedback, enables sharing), well-targeted to audience** | This section has five questions. You need to evaluate how the design of the application help you be more engaged with the functions. |
| 1. Entertainment: Is the app fun/entertaining to use? Does it use any strategies to increase engagement through entertainment (e.g. through gamification)?   - 1 Dull, not fun or entertaining at all - 2 Mostly boring - 3 OK, fun enough to entertain user for a brief time (< 5 minutes) - 4 Moderately fun and entertaining, would entertain user for some time (5-10 minutes total) - 5 Highly entertaining and fun, would stimulate repeat use   Note: (write done if it is not relevant, applicable, or the decision is hard to be made.)__________ | You could judge the entertainment based on whether the application provide you with **amusement or enjoyment** by using some **fun elements** (fun graphics, fun videos or elements of game playing).  The differences between level 3-5 should be judged by the estimated **time** that the fun elements make you entertained. For example, fun graphics may last shorter (maybe less than 5 minutes) than videos (maybe 5-10 minutes) and gamification elements (if the element of game needs to **repeat use** the application to collect some award, it will stimulate repeat use). |
| 2. Interest: Is the app interesting to use? Does it use any strategies to increase engagement by presenting its content in an interesting way?   - 1 Not interesting at all - 2 Mostly uninteresting - 3 OK, neither interesting nor uninteresting; would engage user for a brief time (< 5 minutes) - 4 Moderately interesting; would engage user for some time (5-10 minutes total) - 5 Very interesting, would engage user in repeat use | You could judge the interest based on the way it **presents the content**. This question may be confused with the question on entertainment, but the difference is that this question focus on the format that the app presenting its content. A good example to distinguish the first two questions provided by the tool developer is “A documentary about the disease could be interesting but not fun.”    If it shows content as textbook format, it is not interesting at all (rate as 1).  If it shows content by using animations, metaphors or examples; or it shows the content by using videos/games, it should rate as interesting (rate as 4) or very interesting (rate as 5).  The differences between 3 to 5 could be judged by the **time** that the application could engage you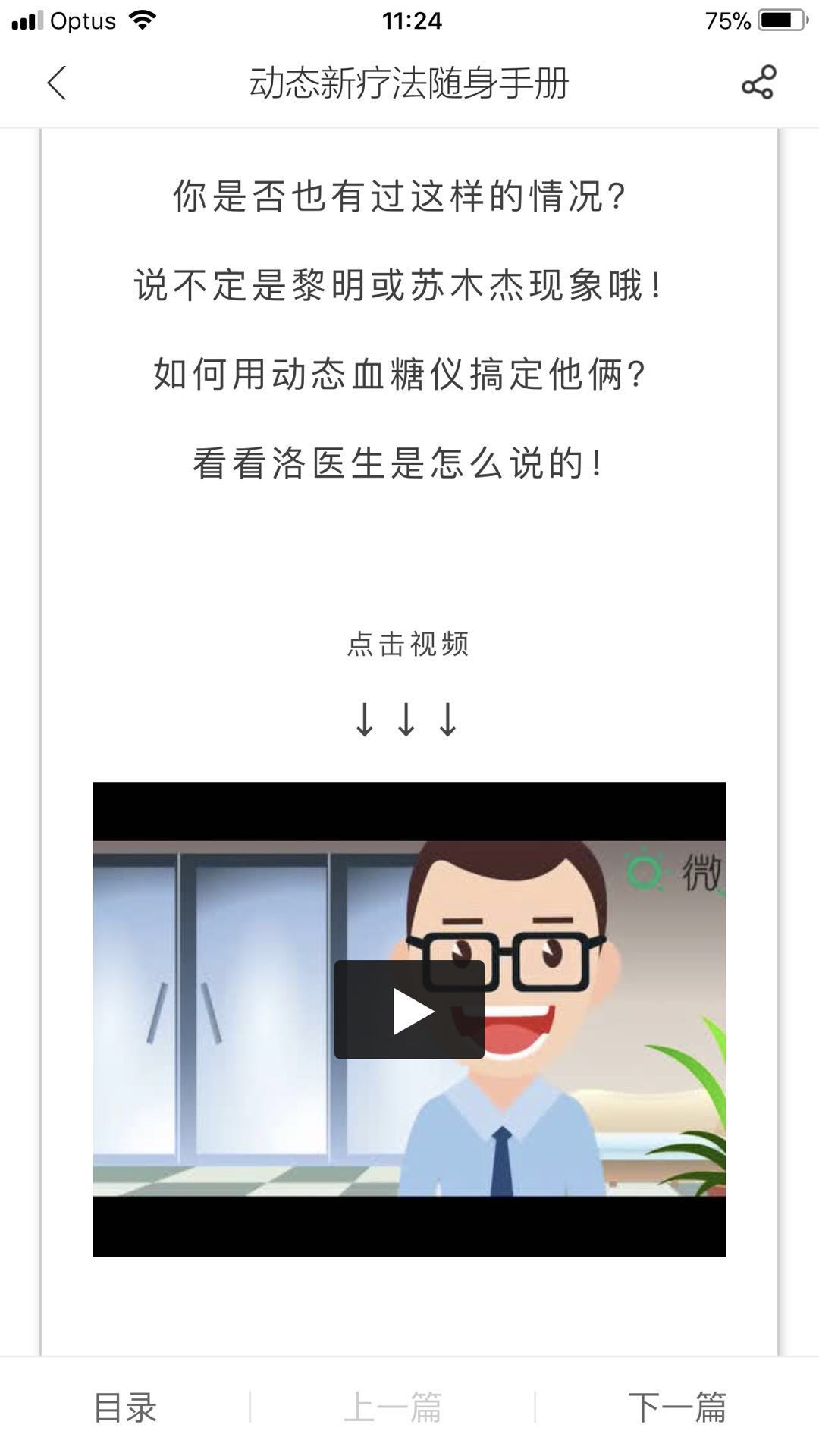 and whether it motivate **repeat use**. |
| 3. Customization: Does it provide/retain all necessary settings/preferences for apps features (e.g.  sound, content, notifications, etc.)?   - 1 Does not allow any customization or requires setting to be input every time - 2 Allows insufficient customization limiting functions - 3 Allows basic customization to function adequately - 4 Allows numerous options for customization - 5 Allows complete tailoring to the individual’s characteristics/preferences, retains all settings | This question could be judged by whether you could **change the settings** of the sound, content, notifications based on your preferences.  If there is a setting button that you could change the setting, you should at least rate as 3. If there are numerous options for customization, then rate as 4. Rate 5 need to be more focused on “individualization”, which means you can not only turn on/off some function (notification, sound) but also change it based on your own preference (sound level, vibration, own music etc) or your preferred content (notification on asthma symptoms, medication use, physical activity, etc.) |
| 4. Interactivity: Does it allow user input, provide feedback, contain prompts (reminders, sharing options, notifications, etc.)? Note: these functions need to be customizable and not overwhelming in order to be perfect.   - 1 No interactive features and/or no response to user interaction - 2 Insufficient interactivities, or feedback, or user input options, limiting functions - 3 Basic interactive features to function adequately - 4 Offers a variety of interactive features/feedback/user input options - 5 Very high level of responsiveness through interactive features/feedback/user input options | This question could be judged by whether the app has features **allows two-way flow of information between you and the content of application information** (whether you could input information, get feedback).  The differences between 3-5 could be judged by the variety of interactive features and the level of responsiveness.  For example, if you could enter the number of puffs of reliever you have taken and get a feedback on whether your asthma is well controlled, then you should at least rate it as 3.  If there are some further instruction/reminder to the user on what to do next, then rate 4. If there is a continuous input-feedback loop (e.g. a check-in of asthma symptoms and technique when noticing that reliver use is high and suggest seeking health advice and/or there seems like a great degree of responsiveness is present) you could rate 5. |
| 5. Target group: Is the app content (visual information, language, design) appropriate for your target audience?   - 1 Completely inappropriate/unclear/confusing - 2 Mostly inappropriate/unclear/confusing - 3 Acceptable but not targeted. May be inappropriate/unclear/confusing - 4 Well-targeted, with negligible issues - 5 Perfectly targeted, no issues found | Specific to our research question, this question could be judged by whether the content (information, language, design) of the app is **appropriate for asthma patients**.  For example, if the terms and language used in the app are too technical to the point where those without medical training cannot easily understand, then it could be rate as 3 or less. If it is well targeted towards asthmatic patients this could be a 4 or 5.  Some apps may have a more specific target groups within the apps (like towards young adults, those using smart devices etc). If the app is well targeted towards one of these subpopulations you could further judge it as a 4-5. |
| 1. **Functionality – app functioning, easy to learn, navigation, flow logic, and gestural design of app** | This section has four questions. You could rate the app mainly based on whether the app could function well and how easy it is to let you learn how to use it. |
| 6. Performance: How accurately/fast do the app features (functions) and components (buttons/menus) work?   - 1 App is broken; no/insufficient/inaccurate response (e.g. crashes/bugs/broken features, etc.) - 2 Some functions work, but lagging or contains major technical problems - 3 App works overall. Some technical problems need fixing/Slow at times - 4 Mostly functional with minor/negligible problems - 5 Perfect/timely response; no technical bugs found/contains a ‘loading time left’ indicator | This question could be judged by **whether or not the app can be used without issues.**  Rate as 3 or higher if the main functions worked. Rate as 4 if there is only 1-2 minor bugs or it runs a little slow only on some function. Rate as 5 if there is no technical bugs and it could run fast. |
| 7. Ease of use: How easy is it to learn how to use the app; how clear are the menu labels/icons and instructions?   - 1 No/limited instructions; menu labels/icons are confusing; complicated - 2 Useable after a lot of time/effort - 3 Useable after some time/effort - 4 Easy to learn how to use the app (or has clear instructions) - 5 Able to use app immediately; intuitive; simple | This question could be judged by how **intuitive** the app is and **how easy you could learn how to use the app**.  Rate 5, if you could immediately understand and use the app. Rate 1, if you are not able to know how to use because of unclear buttons or no tutorial.  Ratings between 2-4 could be decided by the time you spend in understanding the app. Rate 4, if you could learn how to use the app within 10 minutes; Rate 3, if you spent 10-20 minutes; Rate 2 if you spent more than 20 minutes to understand how to use the app. |
| 8. Navigation: Is moving between screens logical/accurate/appropriate/ uninterrupted; are all necessary screen links present?   - 1 Different sections within the app seem logically disconnected and random/confusing/navigation is difficult - 2 Usable after a lot of time/effort - 3 Usable after some time/effort - 4 Easy to use or missing a negligible link - 5 Perfectly logical, easy, clear and intuitive screen flows throughout, or offers shortcuts | This question could be judged by how **easy and accurate the app is to navigate** between pages.  Rate 5 if every button works and is intuitive/logical with shortcuts. For a 5 apps should not only be laid out in a logical way with a clear path to your destination in the app, but also give you the ability to use/create shortcuts if you want to jump into certain page directly.  Rate 1 if the navigation failed in some functions (missing button or link on key pages).  Rating between 2-4 could be decided by the time you spend and how well the navigation goes. Rate 3 if you spend 10-20 minutes to understand the logical flow. |
| 9. Gestural design: Are interactions (taps/swipes/pinches/scrolls) consistent and intuitive across all components/screens?   - 1 Completely inconsistent/confusing - 2 Often inconsistent/confusing - 3 OK with some inconsistencies/confusing elements - 4 Mostly consistent/intuitive with negligible problems - 5 Perfectly consistent and intuitive | Gestural design includes **the design of the figure gesture** (tap or double tap the buttons, swipes the screen from left to right, scroll the page from top to bottom, etc.).  This question will be judged by whether the design of these gesture follows a **consistent way** across all screens and with **enough intuitive information** so that users could easily understand how to use the app.  Rate 5 if you didn’t find any problem  Rate 4 if you find some inconsistency/there is no instruction on certain buttons necessitating 1-2 minutes to figure it out.  Rate 3 if you found some confusing elements but in general it is ok. |
| **C. Aesthetics – graphic design, overall visual appeal, color scheme, and stylistic consistency** | This section mainly focusses on the visual design of the app. **Please make sure that you do not add any personal preference when you rate it.** The general rule is whether the visual design is clear, looks professional and **appeal to our target population (asthma patients).** |
| 10. Layout: Is arrangement and size of buttons/icons/menus/content on the screen appropriate or zoomable if needed?   - 1 Very bad design, cluttered, some options impossible to select/locate/see/read device display not optimized - 2 Bad design, random, unclear, some options difficult to select/locate/see/read - 3 Satisfactory, few problems with selecting/locating/seeing/reading items or with minor screen size problems - 4 Mostly clear, able to select/locate/see/read items - 5 Professional, simple, clear, orderly, logically organized, device display optimized. Every design component has a purpose | This question focuses on the way in which text/buttons/pictures are set out on a page.  A perfect layout means simple, clear, easy to follow and looks professional.  The layout that could be rated as 5 would be one with a reasonable sized font (e.g. with a large title and smaller content), meaningful icons and a well-organized page based on a certain logic and consistency in style.  If there is too much information within a page and the font of words are too small to read, it is a very bad design (rate 1).  The range between 2-4 could be decided by the number of problematic pages and the degree of the problem. |
| 11. Graphics: How high is the quality/resolution of graphics used for buttons/icons/menus/content?   - 1 Graphics appear amateur, very poor visual design - disproportionate, completely stylistically inconsistent - 2 Low quality/low resolution graphics; low quality visual design – disproportionate, stylistically inconsistent - 3 Moderate quality graphics and visual design (generally consistent in style) - 4 High quality/resolution graphics and visual design – mostly proportionate, stylistically consistent - 5 Very high quality/resolution graphics and visual design - proportionate, stylistically consistent throughout | This question focuses on **the quality/resolution/visual design of the graphs**.  The differences between level 4-5 is based on whether most of graphs are in high quality and resolution or all graphs are in high quality.  Level 3 means an acceptable level that graphics are in moderate quality and the moderate quality will not affect the use of the app.  Level 1-2 means low quality and poor visual design. |
| 12. Visual appeal: How good does the app look?   - 1 No visual appeal, unpleasant to look at, poorly designed, clashing/mismatched colours - 2 Little visual appeal – poorly designed, bad use of colour, visually boring - 3 Some visual appeal – average, neither pleasant, nor unpleasant - 4 High level of visual appeal – seamless graphics – consistent and professionally designed - 5 As above + very attractive, memorable, stands out; use of colour enhances app features/menus | This question focuses on whether the visual design of the pages looks appealing.  Please noted that “colorful” doesn’t mean good all the time, but the colours and icons should be consistent and be able to support the functions.  The difference between a 4-5 is that for a 5 the visual appeal enhanced the features of the app itself and adds to the overall enjoyment/use of the program. |
| 1. **Information – Contains high quality information (e.g. text, feedback, measures, references) from a credible source. Select N/A if the app component is irrelevant.** | This section contains 7 questions focus on the quality of the information that the App contains. Five questions in this section has an N/A choice, if the question is irrelevant. |
| 13. Accuracy of app description (in app store): Does app contain what is described?   - 1 Misleading. App does not contain the described components/functions. Or has no description - 2 Inaccurate. App contains very few of the described components/functions - 3 OK. App contains some of the described components/functions - 4 Accurate. App contains most of the described components/functions - 5 Highly accurate description of the app components/functions | This question should be judged by **whether the App contains components and functions as what is described in the APP store**. Therefore, you must read the description of the app in the app store carefully before rating. |
| 14. Goals: Does app have specific, measurable and achievable goals (specified in app store description or within the app itself)?   - N/A Description does not list goals, or app goals are irrelevant to research goal (e.g. using a game for educational purposes) - 1 App has no chance of achieving its stated goals - 2 Description lists some goals, but app has very little chance of achieving them - 3 OK. App has clear goals, which may be achievable. - 4 App has clearly specified goals, which are measurable and achievable - 5 App has specific and measurable goals, which are highly likely to be achieved | This question should be judged by **whether the App (information in the app store) has described any goals** of the app and **whether such goal is achievable through using this App.**  If the description in App store and within the App does not state any goal, please choose N/A.  If there are goals stated, the rating will be decided whether the goal is measurable and achievable.  For example, if the goal is to manage exacerbation of asthmas and the app only provides information on health education, then the goal cannot be measured through the app and is hard to achieve. If the app could record asthma symptoms, provide reminders for using preventers and direction to asthma action plans, then the goal of managing asthma exacerbations is measurable and likely to be achieved (rated as 4 or 5 based on the comprehensiveness of the app). |
| 15. Quality of information: Is app content correct, well written, and relevant to the goal/topic of the app?   - N/A There is no information within the app - 1 Irrelevant/inappropriate/incoherent/incorrect - 2 Poor. Barely relevant/appropriate/coherent/may be incorrect - 3 Moderately relevant/appropriate/coherent/and appears correct - 4 Relevant/appropriate/coherent/correct - 5 Highly relevant, appropriate, coherent, and correct | This question focuses on the **quality of the information (whether the information is relevant, scientific and well written)**. *Reviewers must have read the supporting materials and have a basic understanding about asthma self-management before the rating*. Please read the information in the app and make the judgement about whether the information is relevant, and scientific sound.  Rate 5 if you don’t find any information inappropriate or incorrect. |
| 16. Quantity of information: Is the extent coverage within the scope of the app; and comprehensive but concise?   - N/A There is no information within the app - 1 Minimal or overwhelming - 2 Insufficient or possibly overwhelming - 3 OK but not comprehensive or concise - 4 Offers a broad range of information, has some gaps or unnecessary detail; or has no links to more information and resources - 5 Comprehensive and concise; contains links to more information and resources | This question focuses on the **quantity of the information** of the app. **Insufficient** or **overwhelming** are two extremes.  **Comprehensiveness** could be judged by the scope of the information coverage: for example, if the app covers how to prevent and manage asthma symptoms and exacerbations or manage common asthma co-morbidities, we could give a rating of 4-5 as it is comprehensive. The differences between 4 and 5 is whether the app could link to more information and resources external to the application.  If the app provides too little information (insufficient) or too much information on one single topic (overwhelming for a person not from a health background and not concise enough), you could rate as three or below. |
| 17. Visual information: Is visual explanation of concepts – through charts/graphs/images/videos, etc. – clear, logical, correct?  N/A There is no visual information within the app (e.g. it only contains audio, or text)   - 1 Completely unclear/confusing/wrong or necessary but missing - 2 Mostly unclear/confusing/wrong - 3 OK but often unclear/confusing/wrong - 4 Mostly clear/logical/correct with negligible issues - 5 Perfectly clear/logical/correct | This question focuses on whether the **visual information** provided through charts/graphs/images/videos is **clear and correct**.  Choose NA if there is no icon, graphs, images or videos. The level could be judged by the **proportion of graphs** which are in perfect shape. Rating 5 if all graphs are clear and correct. Rating 4 if most graphic looks fine. Rating 3 if about half are clear.  A confusing and irrelevant graph or image for the intended demographic, such as the image of a flow-volume loop would score a rating of one, while a well presented logical and correct step by step graphic of how to use a spacer would be scored a 5. |
| 18. Credibility: Does the app come from a legitimate source (specified in app store description or within the app itself)?   - 1 Source identified but legitimacy/trustworthiness of source is questionable (e.g. commercial business with vested interest) - 2 Appears to come from a legitimate source, but it cannot be verified (e.g. has no webpage) - 3 Developed by small NGO/institution (hospital/center, etc.) /specialized commercial business, funding body - 4 Developed by government, university or as above but larger in scale - 5 Developed using nationally competitive government or research funding (e.g. Australian Research Council, NHMRC) | This question focused on the **source of the information as well as the qualification of the development team**. This is mainly based on the information provided on the App store about the **developer**. |
| 19. Evidence base: Has the app been trialled/tested; must be verified by evidence (in published  scientific literature)?   - N/A The app has not been trialled/tested - 1 The evidence suggests the app does not work - 2 App has been trialled (e.g., acceptability, usability, satisfaction ratings) and has partially positive outcomes in studies that are not randomized controlled trials (RCTs), or there is little or no contradictory evidence. - 3 App has been trialled (e.g., acceptability, usability, satisfaction ratings) and has positive outcomes in studies that are not RCTs, and there is no contradictory evidence. - 4 App has been trialled and outcome tested in 1-2 RCTs indicating positive results 5 App has been trialled and outcome tested in > 3 high quality RCTs indicating positive results | This question should be judged by **whether the App has been tested**. To answer this question, please search the app name through google scholar or other literature database (pubmed, embase etc.).  Choose N/A if you found no information.  If the app was used in a trial, you should read the related article carefully especially about the study design (whether it is a randomized controlled trial), the outcome values (acceptability, usability, satisfaction, effectiveness, etc.) to make an informed decision. |
| **E. subjective quality** | From this section, you could rate the app based on **your experience of using this app**. |
| 20. Would you recommend this app to people who might benefit from it?   - 1 Not at all I would not recommend this app to anyone - 2 There are very few people I would recommend this app to - 3 Maybe There are several people whom I would recommend it to - 4 There are many people I would recommend this app to - 5 Definitely I would recommend this app to everyone | Specific to our project, please answer the question as would you recommend this app to people with Asthma? |
| 21. How many times do you think you would use this app in the next 12 months if it was relevant to you?   - 1 None - 2 1-2 - 3 3-10 - 4 10-50 - 5 >50 | Specific to our project, please answer the question as if you were an asthmatic patient. How many times do you think you would use this app in the next 12 months? |
| 22. Would you pay for this app?   - 1 No - 3 Maybe - 5 Yes | Specific to our project, answer as if you were an asthmatic patient. Will you pay for this app? |
| 23. What is your overall star rating of the app?   - 1 one star, one of the worst apps I’ve used - 2 two stars - 3 three stars, Average - 4 four stars - 5 five stars, one of the best apps I've used |  |
| **Section F App specific: These added items can be adjusted and used to assess the perceived impact of the app on the users’ knowledge, attitudes, intentions to change as well as the likelihood of actual change in the target health behaviour** | The following questions asked you to evaluate how this app may **influence users’ knowledge, attitude and behaviours**. Specific to our project, all questions were specified to asthma self-management.  All questions should be answered based on how you agree with the statement by using **five-Likert agreement scale from strongly disagree to strongly agree.**  Please read the supporting articles related to behaviour change and asthma self-management as references. |
| Awareness: This app is likely to increase awareness of the importance of addressing asthma self-management. | By using this App, will the users know the fact that self-management of asthma is important? |
| Knowledge: This app is likely to increase knowledge/understanding of asthma self-management. | By using this App, will the users know more about asthma itself and how they should do to better manage their conditions? |
| Attitudes: This app is likely to change attitudes toward improving asthma self-management. | By using this App, will the users regard asthma self-management as positive and meaningful? |
| Intention to change: This app is likely to increase intentions/motivation to address asthma. | By using this App, will the users have a stronger intention to make some changes in their life to better manage their asthma? |
| Help seeking: Use of this app is likely to encourage further help seeking for asthma management. | By using this App, will the users be encouraged to seek help from certain sources for managing their asthma? |
| Behaviour change: Use of this app is likely increase the management of asthma. | By using this App, will the users really change their behaviours (including all relevant behaviours, such as better adherence to treatment, physical activity, identification of exacerbations, proper inhaler technique etc.) |

### Presence of App Features consistent with Asthma Guidelines

#### 4.1 Asthma Education/Knowledge

- To answer these questions appropriately you will need a baseline understanding of asthma and asthma management. This will be achieved through a training session with the project lead and, more importantly, reading the articles provided prior to reviewing the apps.
- **Provides knowledge on:** This is a simple dichotomous yes or no question. If an application provides knowledge on the topic it gets a yes, if not it is a no. This question does not ask about the quality of said information
- **Knowledge is individualised:** The concept of individualisation is key in self-management of chronic conditions. It is one thing to list the symptoms or treatment options of asthma but another to target this to the patient. Individualised information would be an application that asks what symptoms a person experiences and then explains to them why/how these are symptoms of asthma. It could also direct the patient to their action plan to explain the early treatment of asthma. You may find that it is sometimes difficult to conceptualise how a particular piece of knowledge could be individualised. For example, “importance of not only using SABA therapy” could be seen as difficult to individualise. However, all of this information can be individualised by taking the person’s experiences and specific circumstances into account. I suggest you read these and think about ways you as a clinician could personalise this information for patients. Individualising this information will be something we discuss in the training session.
- **The knowledge is evidence based:** Is the information consistent with the resources you have been provided? Is it consistent with what you have been taught about asthma? Does it have reputable references attached to it? These would be questions to consider when thinking if knowledge is evidence based or not.
- **Breadth of knowledge provided:** This is to give us a general idea of what degree of knowledge this app provides for each topic. Add up the “yeses” from the topic and divide it by the number of subtopics. This will give us a percentage. If <50% of the topics are covered this is a poor breadth of knowledge, 50-80% is good and >80% is exceptional.

#### 4. 2 Provides skill training for asthma self-management

- Skill training is key to empower patients to use the equipment that they have in an appropriate manner to manage their asthma. Many people do not use a spacer, many improperly use inhalers and many do not understand when to refer to their asthma action plan. This area is to look at how does the application provide training in these areas. Again, information from this will come from the training session and from the articles you are required to read prior to commencing the data collection.
- **General Skill training:** Would for example demonstrate a generic peak flow meter, how to use it and what general values would be considered poor. For inhaler device use this would refer to generic inhalers, spacers etc. For non-pharmacological strategies this would be general advice such as “avoid triggers like x, y, z”.
- **Personalised skill training:** For this personalised training the application should identify what device the patient uses and describe how to use this. The application should specifically refer to the patient’s peak flow device and their peak flow value of concern. For exacerbations this should specifically refer to the symptoms that the patient experiences in an exacerbation, directs them to their specific asthma action plan and refers to known personal triggers of the patient etc.

#### 4.3 Tracking and displaying health information.

- This area is to focus on how well the application captures data related to a patient’s asthma symptoms and management. Information in the required reading will help you to revise what asthma symptoms area. We will touch on some of these in the training session too.
- **The App could support for tracking and recording data on:** Another dichotomous question. Does it ask the patient “have they experienced x, y, z” (e.g. wheeze, cough, chest tightness) as an example of asthma symptoms? Answer yes if it supports tracking of this information and no if it does not.
- **What approach is the data collected:** Please select all that apply. Does the person have to manually input how many puffs of their SABA that they have required or is the app linked to a smart sensor that uploads it from the device?
- **Are there tables/graphs displaying trends/analysis results:** Does the App convert the information that the patient has into a summary graph for easy viewing? Does the App go one step further and analyse the information for the patient? An example of this might be the App analysing increased symptomology and alerting the patient that they may be having an exacerbation of asthma and should seek health advice.
- **Can this information be easily exported i.e. through email to healthcare providers?:** Can the data be emailed to healthcare providers/others? Can it be uploaded to the MyHealth record or another service?
- **These features are able to be individualised:** Can the user change settings on data collection e.g. what SABA is used, what symptoms are tracked etc. This does not refer to settings that alter the technical ability of the application such as frequency of recording health information or font size of data etc. This type of information is collected in the MARS framework. Here we want to capture the health information. If there the tracking ability cannot be individualised, select no.

#### 4.4 The App provides prompts on.

- This section is about does the application provide reminders on certain things and can this be individualised? You may need to give the application overnight/24hrs to see these reminders. Please fully explore the App to check if reminders are a function.
- **Reminder on:** This is a dichotomous question. Either it does or does not provide prompts on these sections.
- **The reminders are individualised:** This can mean that the person can set what symptoms they get for asthma, their doctor’s information, what preventer they are on etc. It does not refer to the technical settings in the app that allow you to change reminder settings or sound effects etc. This should be captured in the MARS framework. In this section we want the health information.
